# Draft Genome Sequence of a Strain of *Bacillus intestinalis* sp. nov., a New Member of Sporobiota Isolated from the Small Intestine of a Single Patient with Intestinal Cancer

**DOI:** 10.1128/genomeA.00489-17

**Published:** 2017-06-01

**Authors:** Victor Tetz, George Tetz

**Affiliations:** Human Microbiology Institute, New York, New York, USA

## Abstract

We report here the draft genome sequence of *Bacillus intestinalis* strain 1731, a novel spore-forming bacterium isolated from the small intestine of a patient with intestinal cancer. The genome comprised 4,047,276 bp, with 43.9% G+C content. There were 3,913 predicted protein-coding genes, including those associated with antibiotic resistance and virulence.

## GENOME ANNOUNCEMENT

*Bacillus intestinalis* strain 1731 is an aerobic, spore-forming, motile, Gram-positive, rod-shaped bacterium that was isolated from the small intestine of a patient with intestinal malignancy. The members of the *Bacillaceae* family are well-known representatives of the human gut microbiota and are implicated in a number of pathologies (1–3). Using combined culture and genetic workflow, we have previously identified the unexplored diversity of endospore-forming bacteria within humans; this identification necessitated the characterization of spore-forming bacteria, such as *Sporobiota* spp., and the collection of their genes as a sporobiome, owing to their unique common characteristics (4–6).

The 16S rRNA gene sequences of *B. intestinalis* strain 1731 shared 99% similarity with those of various *Bacillus* strains, including *B. subtilis*, *B. amyloliquefaciens*, and *B. atrophaeus*.

An *in silico* DNA-DNA hybridization (DDH) analysis, using the genome-to-genome distance calculator (GGDC2.1) algorithm, produced a highest DDH value of 50.80%, which indicates that *B. intestinalis* strain 1731 is a new species belonging to the *Bacillus* genus (7).

The genome of *B. intestinalis* strain 1731 was sequenced using an Illumina HiSeq sequencing platform (GA IIx; Illumina, CA). Library preparation, sequencing, and runs were performed in accordance with the manufacturer’s instructions. *De novo* assembly was performed with SPAdes version 3.9.0 (8), and annotation was performed via the NCBI and RAST servers (9, 10). The final draft genome assembly consisted of 164 contigs and 4,047,276 bp (G+C content of 43.9%), and the total coverage over the genome was 145-fold. It comprises 3,913 gene-coding sequences and 106 predicted RNA genes (86 tRNA, 15 rRNA, and 5 ncRNA genes). Genome analysis using the NCBI and CARD databases revealed genes coding for resistance to the antibiotics tunicamycin, fosfomycin, and bleomycin and class A and B beta-lactamases; the genes *mprF*, *aadK*, and *tmrB* (determinant of resistance to peptide, aminoglycoside, and nucleoside antibiotics, respectively); major facilitator superfamily (MFS) transporters; and the organic hydroperoxide resistance protein OhrA (11).

The genome contains virulence factors, including hemolysin D, serine protease, zinc metalloprotease HtpX, peptidases, alpha-amylase, amidohydrolase, phospholipase, phenolic acid decarboxylase, and subtilosin A exodeoxyribonuclease III and VII capsular, flagellar, and sporulation proteins (12, 13). In addition, we identified superoxide dismutases that are considered to possess carcinogenic properties in some bacteria (14).

The availability of the *B. intestinalis* genome sequence facilitates further analysis of human sporobiota and its possible implications in intestinal malignization.

### Accession number(s).

The complete genome sequence has been deposited in the NCBI database under the accession no. MWZB00000000.
